# *N*-(3-Chloro-4-methyl­phen­yl)maleamic acid

**DOI:** 10.1107/S1600536812007842

**Published:** 2012-02-29

**Authors:** U. Chaithanya, Sabine Foro, B. Thimme Gowda

**Affiliations:** a Department of Chemistry, Mangalore University, Mangalagangotri 574 199, Mangalore, India; b Institute of Materials Science, Darmstadt University of Technology, Petersenstrasse 23, D-64287 Darmstadt, Germany

## Abstract

In the title compound, C_11_H_10_ClNO_3_, the dihedral angle between the benzene ring and the amide group is 6.6 (10)° and an intramolecular O—H⋯O hydrogen bond occurs. In the crystal, molecules are linked by N—H⋯O hydrogen bonds, generating *C*(7) zigzag chains.

## Related literature

For our studies on the effects of substituents on the structures and other aspects of *N*-(ar­yl)-amides, see: Gowda *et al.* (2000 ▶, 2003 ▶, 2007 ▶); Chaithanya *et al.* (2012 ▶). For *N*-chloro­aryl­amides, see: Jyothi & Gowda (2004 ▶). For *N*-bromo­aryl­sulfonamides, see: Usha & Gowda (2006 ▶).
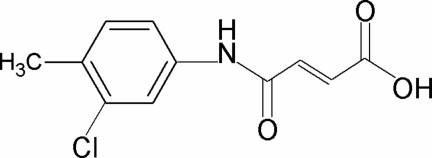


## Experimental

### 

#### Crystal data

C_11_H_10_ClNO_3_
*M* *_r_* = 239.65
Monoclinic, 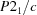

*a* = 9.005 (1) Å
*b* = 13.491 (2) Å
*c* = 8.757 (1) Å
β = 97.91 (1)°
*V* = 1053.7 (2) Å^3^
*Z* = 4
Mo *K*α radiation
μ = 0.35 mm^−1^
*T* = 293 K
0.48 × 0.40 × 0.34 mm

#### Data collection

Oxford Diffraction Xcalibur diffractometer with a Sapphire CCD detector
Absorption correction: multi-scan (*CrysAlis RED*; Oxford Diffraction, 2009 ▶) *T*_min_ = 0.849, *T*_max_ = 0.890
4069 measured reflections
2147 independent reflections
1798 reflections with *I* > 2σ(*I*)
*R*_int_ = 0.011

#### Refinement

*R*[*F* ^2^ > 2σ(*F* ^2^)] = 0.037
*wR*(*F* ^2^) = 0.105
*S* = 1.04
2147 reflections
152 parameters
2 restraints
H atoms treated by a mixture of independent and constrained refinement
Δρ_max_ = 0.33 e Å^−3^
Δρ_min_ = −0.27 e Å^−3^

### 

Data collection: *CrysAlis CCD* (Oxford Diffraction, 2009 ▶); cell refinement: *CrysAlis RED* (Oxford Diffraction, 2009 ▶); data reduction: *CrysAlis RED*; program(s) used to solve structure: *SHELXS97* (Sheldrick, 2008 ▶); program(s) used to refine structure: *SHELXL97* (Sheldrick, 2008 ▶); molecular graphics: *PLATON* (Spek, 2009 ▶); software used to prepare material for publication: *SHELXL97*.

## Supplementary Material

Crystal structure: contains datablock(s) I, global. DOI: 10.1107/S1600536812007842/kp2391sup1.cif

Structure factors: contains datablock(s) I. DOI: 10.1107/S1600536812007842/kp2391Isup2.hkl

Supplementary material file. DOI: 10.1107/S1600536812007842/kp2391Isup3.cml

Additional supplementary materials:  crystallographic information; 3D view; checkCIF report

## Figures and Tables

**Table 1:** Hydrogen-bond geometry (Å, °)

*D*—H⋯*A*	*D*—H	H⋯*A*	*D*⋯*A*	*D*—H⋯*A*
N1—H1N⋯O2^i^	0.87 (2)	2.11 (2)	2.9546 (19)	164 (2)
O3—H3O⋯O1	0.87 (2)	1.62 (2)	2.4885 (17)	173 (2)

Symmetry code: (i) 
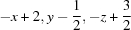
.

**Fig. 1.**
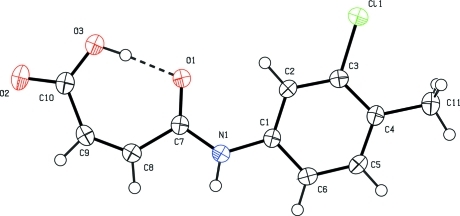
Molecular structure of the title compound showing the atom labelling scheme with displacement ellipsoids drawn at the 50% probability level. Intramolecular hydrogen bond in shown.

**Fig. 2.**
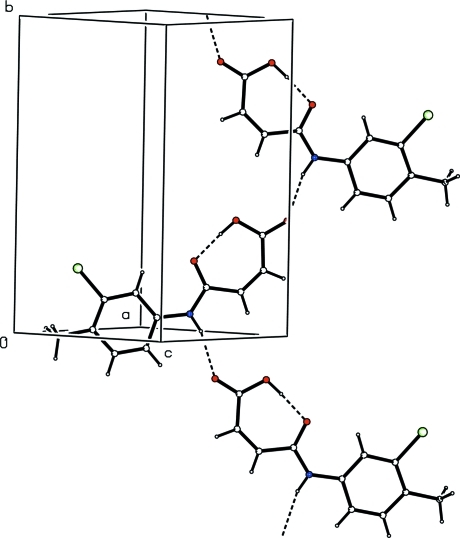
Molecular packing of the title compound with hydrogen bonding shown as dashed lines.

## supplementary crystallographic information

### Comment

As part of our studies on the substituent effects on the structures and other aspects of *N*-(aryl)-amides (Gowda *et al.*, 2000, 2003, 2007; Chaithanya *et al.*, 2012), *N*-chloroarylsulfonamides (Jyothi & Gowda, 2004) and *N*-bromoarylsulfonamides (Usha & Gowda, 2006), in the present work, the crystal structure of *N*-(3-chloro-4-methylphenyl)maleamic acid has been determined (Fig. 1). The conformations of the N—H and the C=O bonds in the amide segment are *anti* to each other. The conformation of the N—H bond is also *anti* to the *meta*–chloro atom. Further, the conformation of the amide C═O is *anti* to the H atom on the adjacent –CH group, while the carboxyl C═O of the acid segment is *syn* to the adjacent –CH group. Furthermore, the C═O and O—H bond of the acid group are in relatively rare *anti* position to each other, due to the donation of hydrogen bond to the amide by the carboxyl group, in contrast to the more general *syn* conformation observed in *N*-(3-chloro-4-methylphenyl)-succinamic acid (I) (Chaithanya *et al.*, 2012).

The dihedral angle between the phenyl ring and the amide group in the title compound is 6.55 (99)°, compared to the values of 40.58 (22)° and 44.93 (27)° in the two derivatives of (I).

In the structure, the pairs of O—H···O and N—H···O intermolecular hydrogen bonds pack the molecules into chains (Table 1, Fig.2).

### Experimental

Maleic anhydride (0.025 mol) in toluene (25 mL) was treated dropwise with 3-chloro-4-methylaniline (0.025 mol) also in toluene (20 mL) with constant stirring. The resulting mixture was stirred for about 30 min and set aside for an additional 30 min at room temperature for the completion of reaction. The mixture was then treated with dilute hydrochloric acid to remove the unreacted 3-chloro-4-methylaniline. The resultant solid *N*-(3-chloro-4-methylphenyl)-maleamic acid was filtered under suction and washed thoroughly with water to remove the unreacted maleic anhydride and maleic acid. It was recrystallised to constant melting point from ethanol. The purity of the compound was checked and characterized by its infrared spectra.

Prism like pale yellow single crystals of the title compound used in X-ray diffraction studies were grown in an ethanol solution by slow evaporation of the solvent (0.5 g in about 30 mL of ethanol) at room temperature.

### Refinement

The H atoms of the NH group and the OH group were located in a difference map and later restrained to the distance N—H = 0.86 (2) Å and O—H = 0.82 (2) Å, respectively. The other H atoms were positioned with idealized geometry using a riding model with the aromatic C—H = 0.93 Å and methylene C—H = 0.97 Å. All H atoms were refined with isotropic displacement parameters set at 1.2 *U*_eq_(C-aromatic, N) and 1.5 *U*_eq_(*C*-methyl).

### Crystal data

**Table:**

C_11_H_10_ClNO_3_	*F*(000) = 496
*M**_r_* = 239.65	*D*_x_ = 1.511 Mg m^−^^3^
Monoclinic, *P*2_1_/*c*	Mo *K*α radiation, λ = 0.71073 Å
Hall symbol: -P 2ybc	Cell parameters from 1538 reflections
*a* = 9.005 (1) Å	θ = 2.8–27.9°
*b* = 13.491 (2) Å	µ = 0.35 mm^−^^1^
*c* = 8.757 (1) Å	*T* = 293 K
β = 97.91 (1)°	Prism, yellow
*V* = 1053.7 (2) Å^3^	0.48 × 0.40 × 0.34 mm
*Z* = 4	

### Data collection

**Table:**

Oxford Diffraction Xcalibur diffractometer with a Sapphire CCD detector	2147 independent reflections
Radiation source: fine-focus sealed tube	1798 reflections with *I* > 2σ(*I*)
Graphite monochromator	*R*_int_ = 0.011
Rotation method data acquisition using ω and phi scans	θ_max_ = 26.4°, θ_min_ = 2.8°
Absorption correction: multi-scan (*CrysAlis RED*; Oxford Diffraction, 2009)	*h* = −11→8
*T*_min_ = 0.849, *T*_max_ = 0.890	*k* = −16→14
4069 measured reflections	*l* = −7→10

### Refinement

**Table:**

Refinement on *F*^2^	Primary atom site location: structure-invariant direct methods
Least-squares matrix: full	Secondary atom site location: difference Fourier map
*R*[*F*^2^ > 2σ(*F*^2^)] = 0.037	Hydrogen site location: inferred from neighbouring sites
*wR*(*F*^2^) = 0.105	H atoms treated by a mixture of independent and constrained refinement
*S* = 1.04	*w* = 1/[σ^2^(*F*_o_^2^) + (0.0582*P*)^2^ + 0.2934*P*] where *P* = (*F*_o_^2^ + 2*F*_c_^2^)/3
2147 reflections	(Δ/σ)_max_ < 0.001
152 parameters	Δρ_max_ = 0.33 e Å^−^^3^
2 restraints	Δρ_min_ = −0.27 e Å^−^^3^

### Special details

**Table:**

Experimental. CrysAlis RED (Oxford Diffraction, 2009) Empirical absorption correction using spherical harmonics, implemented in SCALE3 ABSPACK scaling algorithm.
Geometry. All e.s.d.'s (except the e.s.d. in the dihedral angle between two l.s. planes) are estimated using the full covariance matrix. The cell e.s.d.'s are taken into account individually in the estimation of e.s.d.'s in distances, angles and torsion angles; correlations between e.s.d.'s in cell parameters are only used when they are defined by crystal symmetry. An approximate (isotropic) treatment of cell e.s.d.'s is used for estimating e.s.d.'s involving l.s. planes.
Refinement. Refinement of *F*^2^ against ALL reflections. The weighted *R*-factor *wR* and goodness of fit *S* are based on *F*^2^, conventional *R*-factors *R* are based on *F*, with *F* set to zero for negative *F*^2^. The threshold expression of *F*^2^ > σ(*F*^2^) is used only for calculating *R*-factors(gt) *etc*. and is not relevant to the choice of reflections for refinement. *R*-factors based on *F*^2^ are statistically about twice as large as those based on *F*, and *R*- factors based on ALL data will be even larger.

### Fractional atomic coordinates and isotropic or equivalent isotropic displacement parameters (Å^2^)

**Table:**

	*x*	*y*	*z*	*U*_iso_*/*U*_eq_	
C1	0.66874 (17)	0.04762 (11)	0.39541 (18)	0.0309 (3)	
C2	0.59803 (18)	0.12392 (11)	0.30833 (19)	0.0359 (4)	
H2	0.6327	0.1887	0.3217	0.043*	
C3	0.47494 (18)	0.10217 (11)	0.20120 (19)	0.0355 (4)	
C4	0.41627 (17)	0.00764 (12)	0.17621 (18)	0.0332 (4)	
C5	0.49161 (19)	−0.06669 (12)	0.2641 (2)	0.0398 (4)	
H5	0.4571	−0.1315	0.2503	0.048*	
C6	0.61566 (18)	−0.04879 (12)	0.3714 (2)	0.0381 (4)	
H6	0.6637	−0.1009	0.4275	0.046*	
C7	0.85614 (17)	0.14437 (12)	0.56761 (18)	0.0324 (3)	
C8	0.98127 (18)	0.13165 (12)	0.69471 (19)	0.0351 (4)	
H8	1.0081	0.0665	0.7197	0.042*	
C9	1.06040 (18)	0.20092 (13)	0.77789 (19)	0.0378 (4)	
H9	1.1357	0.1758	0.8509	0.045*	
C10	1.05105 (18)	0.31142 (12)	0.77605 (19)	0.0366 (4)	
C11	0.27987 (18)	−0.01421 (14)	0.0625 (2)	0.0434 (4)	
H11A	0.2960	0.0081	−0.0380	0.052*	
H11B	0.1949	0.0196	0.0930	0.052*	
H11C	0.2614	−0.0843	0.0598	0.052*	
N1	0.79351 (15)	0.06004 (10)	0.51139 (16)	0.0338 (3)	
H1N	0.827 (2)	0.0067 (12)	0.560 (2)	0.041*	
O1	0.81351 (14)	0.22693 (9)	0.51661 (15)	0.0483 (3)	
O2	1.13005 (15)	0.35781 (10)	0.87433 (15)	0.0507 (4)	
O3	0.95988 (16)	0.35732 (9)	0.67213 (17)	0.0537 (4)	
H3O	0.911 (2)	0.3136 (16)	0.611 (2)	0.064*	
Cl1	0.38990 (6)	0.19890 (3)	0.09113 (6)	0.0620 (2)	

### Atomic displacement parameters (Å^2^)

**Table:**

	*U*^11^	*U*^22^	*U*^33^	*U*^12^	*U*^13^	*U*^23^
C1	0.0306 (7)	0.0240 (7)	0.0350 (8)	−0.0015 (6)	−0.0065 (6)	−0.0020 (6)
C2	0.0396 (8)	0.0203 (7)	0.0427 (9)	−0.0025 (6)	−0.0119 (7)	−0.0008 (6)
C3	0.0381 (8)	0.0234 (7)	0.0407 (9)	0.0018 (6)	−0.0099 (7)	−0.0006 (7)
C4	0.0324 (8)	0.0264 (8)	0.0375 (8)	−0.0021 (6)	−0.0064 (6)	−0.0040 (6)
C5	0.0425 (9)	0.0218 (8)	0.0503 (10)	−0.0058 (6)	−0.0112 (7)	−0.0008 (7)
C6	0.0421 (9)	0.0219 (8)	0.0453 (9)	−0.0008 (6)	−0.0118 (7)	0.0024 (7)
C7	0.0323 (7)	0.0259 (7)	0.0359 (8)	−0.0011 (6)	−0.0064 (6)	−0.0018 (6)
C8	0.0372 (8)	0.0264 (8)	0.0378 (9)	0.0012 (6)	−0.0083 (7)	0.0009 (6)
C9	0.0374 (8)	0.0341 (9)	0.0368 (8)	0.0010 (7)	−0.0125 (7)	0.0008 (7)
C10	0.0370 (8)	0.0322 (8)	0.0376 (8)	−0.0037 (7)	−0.0051 (7)	−0.0042 (7)
C11	0.0395 (9)	0.0356 (9)	0.0497 (10)	−0.0037 (7)	−0.0135 (8)	−0.0050 (8)
N1	0.0354 (7)	0.0229 (6)	0.0385 (7)	−0.0005 (5)	−0.0115 (6)	0.0022 (5)
O1	0.0517 (7)	0.0246 (6)	0.0584 (8)	−0.0014 (5)	−0.0286 (6)	0.0011 (5)
O2	0.0559 (8)	0.0382 (7)	0.0511 (8)	−0.0093 (6)	−0.0174 (6)	−0.0103 (6)
O3	0.0593 (8)	0.0273 (6)	0.0638 (9)	−0.0037 (6)	−0.0298 (6)	−0.0009 (6)
Cl1	0.0708 (4)	0.0261 (2)	0.0743 (4)	0.00032 (19)	−0.0426 (3)	0.0058 (2)

### Geometric parameters (Å, º)

**Table:**

C1—C2	1.383 (2)	C7—N1	1.334 (2)
C1—C6	1.392 (2)	C7—C8	1.481 (2)
C1—N1	1.4170 (19)	C8—C9	1.330 (2)
C2—C3	1.382 (2)	C8—H8	0.9300
C2—H2	0.9300	C9—C10	1.493 (2)
C3—C4	1.386 (2)	C9—H9	0.9300
C3—Cl1	1.7364 (16)	C10—O2	1.212 (2)
C4—C5	1.384 (2)	C10—O3	1.296 (2)
C4—C11	1.500 (2)	C11—H11A	0.9600
C5—C6	1.378 (2)	C11—H11B	0.9600
C5—H5	0.9300	C11—H11C	0.9600
C6—H6	0.9300	N1—H1N	0.869 (15)
C7—O1	1.2410 (19)	O3—H3O	0.871 (16)
			
C2—C1—C6	119.32 (14)	N1—C7—C8	114.71 (13)
C2—C1—N1	124.51 (13)	C9—C8—C7	128.69 (15)
C6—C1—N1	116.16 (13)	C9—C8—H8	115.7
C3—C2—C1	118.80 (14)	C7—C8—H8	115.7
C3—C2—H2	120.6	C8—C9—C10	132.10 (15)
C1—C2—H2	120.6	C8—C9—H9	113.9
C2—C3—C4	123.73 (14)	C10—C9—H9	113.9
C2—C3—Cl1	117.95 (12)	O2—C10—O3	120.32 (16)
C4—C3—Cl1	118.31 (12)	O2—C10—C9	118.75 (16)
C5—C4—C3	115.58 (14)	O3—C10—C9	120.92 (14)
C5—C4—C11	121.39 (14)	C4—C11—H11A	109.5
C3—C4—C11	123.03 (14)	C4—C11—H11B	109.5
C6—C5—C4	122.79 (15)	H11A—C11—H11B	109.5
C6—C5—H5	118.6	C4—C11—H11C	109.5
C4—C5—H5	118.6	H11A—C11—H11C	109.5
C5—C6—C1	119.74 (15)	H11B—C11—H11C	109.5
C5—C6—H6	120.1	C7—N1—C1	128.23 (13)
C1—C6—H6	120.1	C7—N1—H1N	115.1 (13)
O1—C7—N1	122.59 (14)	C1—N1—H1N	116.2 (13)
O1—C7—C8	122.70 (14)	C10—O3—H3O	108.9 (16)
			
C6—C1—C2—C3	0.9 (3)	C2—C1—C6—C5	−1.5 (3)
N1—C1—C2—C3	−178.44 (16)	N1—C1—C6—C5	177.89 (15)
C1—C2—C3—C4	0.6 (3)	O1—C7—C8—C9	3.8 (3)
C1—C2—C3—Cl1	−178.99 (13)	N1—C7—C8—C9	−176.65 (18)
C2—C3—C4—C5	−1.5 (3)	C7—C8—C9—C10	1.3 (3)
Cl1—C3—C4—C5	178.12 (13)	C8—C9—C10—O2	173.92 (19)
C2—C3—C4—C11	178.21 (16)	C8—C9—C10—O3	−5.7 (3)
Cl1—C3—C4—C11	−2.2 (2)	O1—C7—N1—C1	−3.5 (3)
C3—C4—C5—C6	0.9 (3)	C8—C7—N1—C1	177.01 (15)
C11—C4—C5—C6	−178.86 (17)	C2—C1—N1—C7	6.7 (3)
C4—C5—C6—C1	0.6 (3)	C6—C1—N1—C7	−172.69 (16)

### Hydrogen-bond geometry (Å, º)

**Table:**

*D*—H···*A*	*D*—H	H···*A*	*D*···*A*	*D*—H···*A*
N1—H1*N*···O2^i^	0.87 (2)	2.11 (2)	2.9546 (19)	164 (2)
O3—H3*O*···O1	0.87 (2)	1.62 (2)	2.4885 (17)	173 (2)

Symmetry code: (i) −*x*+2, *y*−1/2, −*z*+3/2.
